# RapGene: a fast and accurate strategy for synthetic gene assembly in *Escherichia coli*

**DOI:** 10.1038/srep11302

**Published:** 2015-06-11

**Authors:** Massimiliano Zampini, Pauline Rees Stevens, Justin A. Pachebat, Alison Kingston-Smith, Luis A. J. Mur, Finbarr Hayes

**Affiliations:** 1 Institute of Biological, Environmental and Rural Sciences, Edward Llwyd Building, Aberystwyth University, Aberystwyth SY23 3FG, UK; 2 Faculty of Life Sciences, University of Manchester, Manchester M13 9PL, UK

## Abstract

The ability to assemble DNA sequences *de novo* through efficient and powerful DNA fabrication methods is one of the foundational technologies of synthetic biology. Gene synthesis, in particular, has been considered the main driver for the emergence of this new scientific discipline. Here we describe RapGene, a rapid gene assembly technique which was successfully tested for the synthesis and cloning of both prokaryotic and eukaryotic genes through a ligation independent approach. The method developed in this study is a complete bacterial gene synthesis platform for the quick, accurate and cost effective fabrication and cloning of gene-length sequences that employ the widely used host *Escherichia coli*.

Synthetic biology has emerged in recent years as a new frontier in genetic engineering, quickly developing into an independent discipline that has far-reaching potential in numerous spheres including medicine, chemistry, energy, agriculture, and the environment^1^. This process has been fuelled by the simultaneous development of systems biology and of powerful tools such as next generation sequencing technologies. Two key features render synthetic biology markedly different from traditional genetic engineering: the growing availability of novel and highly effective methods for DNA assembly, and the application of engineering principles to biology, namely standardization, modularity and hierarchical abstraction^2^,^3^. Genetic engineering has mainly focused on the use of restriction enzyme-based strategies. In contrast, procedures adopted in synthetic biology allow for quicker and more extensive genetic manipulation while avoiding the constraints of conventional cloning, e.g., the requirement for copies of the same restriction sites in the cloned DNA and vector or the need for consecutive rounds of cloning to introduce multiple sequence variations.

Synthetic biology is based on the use of highly efficient, reliable and cost-effective standardized DNA assembly technologies. In general these approaches differ widely according to the scale of assembly required for a specific project, i.e., synthetic genes, pathways or genomes^4^,^5^. Situated at the lowest hierarchical level of synthetic biology, gene synthesis is a fundamental technology in which chemically synthesized oligonucleotides are assembled to form gene-length double-stranded DNA fragments. A common feature adopted by many gene fabrication strategies is the use of long homology end sequences to assemble several single or double-stranded DNA molecules into complex constructs^6^,^7^. Ligation independent cloning (LIC) has been a prototype for this approach and exploits the use of double-stranded DNA fragments with long cohesive ends to efficiently generate stable cloning intermediates^8^. PCR-based methods such as polymerase cycling assembly (PCA)^9^, ligase chain reaction (LCR)^10^ and thermodynamically balanced inside out synthesis^11^ are among the most commonly used gene assembly methods to date, although numerous other methods have also been published^12^,^13^. At the higher level of pathway synthesis several techniques have been described that permit assembly of gene-length fragments, frequently referred to as synthons^14^, into extensive cassettes up to several kilobases in length. These methods include splicing by overlap extension^15^, enzymatic assembly of overlapping DNA fragments^16^, BioBrick and BglBricks assembly^17^,^18^, seamless ligation cloning extract (SLiCE)^19^, and sequence and ligation independent cloning (SLIC)^20^. Other proprietary technologies in this group include Gateway cloning and Seamless Cloning and Assembly (Life Technologies), In-fusion™ (Clontech), and CloneEZ® (GenScript). Finally, as the ability to manipulate very lengthy DNA molecules is a limiting factor for *in vitro* technologies, genome synthesis methods have been developed for the assembly of megabase length molecules *in vivo*. Yeast species appear to be the ideal chassis in this context, due to their intrinsic high efficiency in homologous recombination and ability to stably maintain DNA of the length of artificial chromosomes^4^,^21^. Despite impressive advances in DNA assembly methodology, the development of simple, fast and effective approaches for the construction of gene-length fragments in research laboratories remains a priority in synthetic biology^4^,^5^,^6^,^7^,^22^.

Gibson recently described the uptake and assembly of overlapping complementary oligonucleotides and a linear double-stranded vector by the yeast *Saccharomyces cerevisiae*^23^. This elegant approach takes advantage of the ability of this host to take up and recombine oligonucleotides into gene-length sequences. Here we describe RapGene, an analogous gene synthesis platform for synthetic gene assembly in *Escherichia coli*. The technique is a quick, accurate and cost-effective technology that has been successfully tested for the assembly and cloning of prokaryotic and eukaryotic genes. Combined with a modified positive selection vector, RapGene is a potent and efficient stratagem for building gene-length sequences in molecular biology laboratories that employ the widely used *E. coli* platform.

## Results

### pRG1.0 is a positive-selection vector based on the toxic marker *Gata1*

Positive-selection vectors are based on the inducible expression of a counter-selectable toxic marker^24^. Cells transformed with a non-recombinant plasmid fail to grow on media containing the inducer of the toxic gene, thereby eliminating the labour-intensive steps of agarose gel purification and vector dephosphorylation, commonly needed to reduce the number of background colonies. The GST-GATA-1 toxic protein consists of part of the DNA binding domain of the mouse transcription factor GATA-1 fused to glutathione-S-transferase. The fusion protein binds to the origin of replication of the *E. coli* chromosome inducing rapid arrest of the cell cycle^25^. The cloning vector pRG1.0 (Supplementary Fig. S1) is a derivative of the pGATA positive selection vector in which the *GST-Gata1* gene is under the control of an IPTG-inducible promoter^25^. The pRG1.0 vector is modified to permit ligation independent cloning and also to avoid the need for a stop codon or a frame-shift mutation downstream of the insert coding sequence that otherwise could still retain toxicity. In fact, the toxic element is entirely deleted when synthetic sequences are inserted between the flanking NheI and AflII restriction enzyme sites (Supplementary Fig. S1).

### RapGene assembly of a synthetic *aadA1* gene in pRG1.0

**Figure 1:**
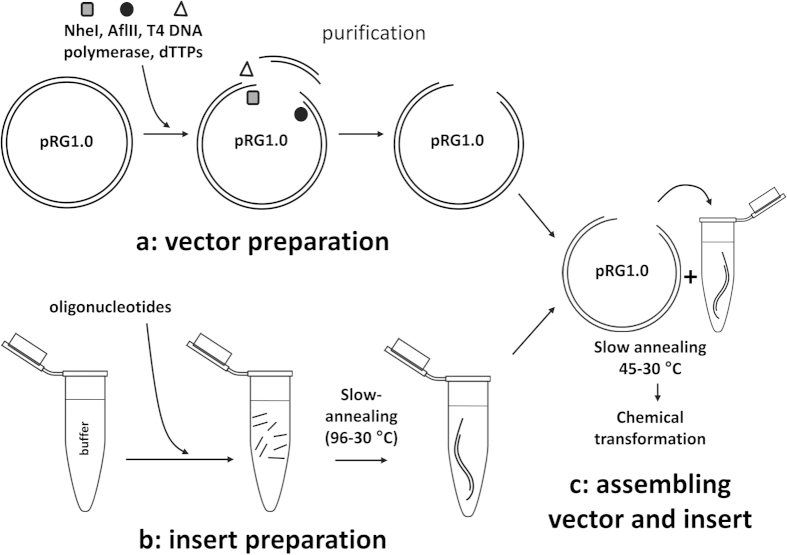
RapGene overview. (**a**) Plasmid pRG1.0 is digested simultaneously with AflII and NheI in the presence of T4 DNA polymerase and dTTPs to generate long 3’-recessed cohesive ends to accommodate the annealed oligonucleotides. (**b**) Oligonucleotides for each gene assembly are pooled in a buffered solution and then subjected to slow annealing (96 °C to 30 °C) for up to two hours. (**c**) Vector and pre-annealed oligonucleotides are further incubated from 45 °C to 30 °C in a 10 μl reaction. This mix is used to transform *E. coli*. Transformations are plated on LB supplemented with ampicillin and IPTG.

The *aadA1* gene encodes spectinomycin resistance in *E. coli*. Full-length (836 bp) and subfragments of *aadA1* were assembled into pRG1.0 by the ligation independent procedure outlined in Methods, and then transformed in *E. coli* NEB5α (Fig. 1). Briefly, pRG1.0 was prepared with long 3’-recessed cohesive ends by simultaneous treatment with AflII, NheI and T4 DNA polymerase to accommodate the annealed oligonucleotides. The full length *aadA1* gene was produced from 28 overlapping, non-phosphorylated oligonucleotides that were 57-60 nt in length. Oligonucleotides Sm-1 to Sm-28 (Supplementary Table S1) that cover the full-length or subfragments of *aad1* were slowly annealed together, incubated with the vector and then introduced into *E. coli* by chemical transformation, as described above. The annealing step was unnecessary when six or fewer oligonucleotides were used and very high yields were observed. Instead ten minutes co-incubation of prepared vector and oligonucleotides at room temperature was sufficient to generate the expected recombinant construct. In this case oligonucleotides were assembled for approx. 5 minutes at room temperature in the buffered solution described in Methods. However, cloning of inserts derived from 24 or more oligonucleotides required the pre-annealing process. After incubation of the prepared vector with the oligonucleotide mix, the reaction was used for transformation of *E. coli*, followed by plating on media containing ampicillin to select for transformants and IPTG to eliminate non-recombinant plasmids that possessed an intact *GST-Gata1* gene. Colonies that grow in the presence of IPTG are expected to possess recombinant pRG1.0 in which the toxic *GST-Gata1* is replaced by the synthetic *aad1* gene.

**Figure 2:**
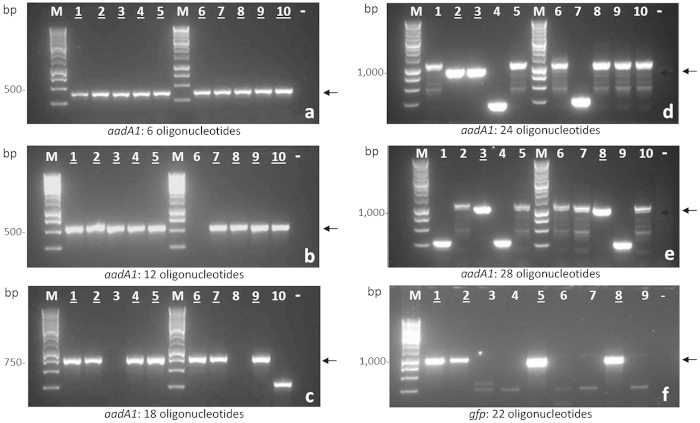
Gel electrophoresis of PCR products derived from amplification of the pRG1.0 region containing synthetic *aad1 or gfp* inserts. (**a**) *aad1* six oligonucleotide assembly. (**b**) *aad1* 12 oligonucleotide assembly. **(c**) *aad1* 18 oligonucleotide assembly. (**d**) *aad1* 24 oligonucleotide assembly. (**e**) *aad1* 28 oligonucleotide assembly. (f) *gfp* 22 oligonucleotide assembly. M is a 1 kb ladder (Promega). Arrows indicate the expected positions of PCR products which were 401 (**a**), 575 (**b**), 749 (c), 923 (**d**), 1040 **(e**) and 965 (**f**) bp. Underlined numbers in each lane represent samples with the correct sized inserts that were also sequence verified. Lanes labelled – are negative controls.

**Figure 3:**
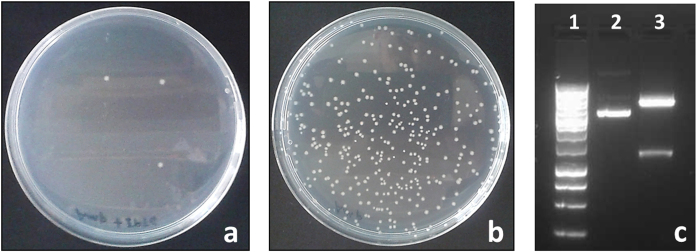
Effect of the positive selection marker on RapGene cloning. pRG1.0 was digested for two hours at 37 °C with AflII and NheI in the presence of T4 DNA polymerase and 1 mM dTTPs. After purification the prepared vector, with no insert, was transformed in *E. coli* NEB5α, SOC was added and cells were incubated for one hour at 37 °C, then one-fifth of the transformation was plated on LB supplemented with ampicillin plus IPTG (**a**) or ampicillin only (**b**). (**c**) Restriction profile of pRG1.0 used for this cloning. Lanes: 1, 1 kb ladder (Promega); 2, pRG1.0 undigested; 3, pRG1.0 digested with AflII, NheI and treated with T4 DNA polymerase in the presence of dTTPs.

When shorter (≤18) oligonucleotide assemblies corresponding to subfragments of *aad1* were used, transformation typically produced >1.6 × 10^5^ to 1.4 ± 2 × 10^3^ CFU/ μg input DNA on LB agar plates supplemented with ampicillin and IPTG (Table 1). Plasmids from these shorter oligonucleotide assemblies were screened by colony PCR, and most carried an insert of the correct size (Fig. 2a–c and Table 1). Subsequently, up to ten recombinant plasmids for each oligonucleotide assembly among those carrying an insert of apparent correct size were sequenced (Table 1). Throughout the six oligonucleotide assemblies 90% of samples were fully correct. In comparison, the inserts derived from 12 and 18 oligonucleotides had sequence accuracies of 70% and 30%, respectively. As a negative control, the vector without an insert produced few colonies (1.5 ± 1 × 10^1^ CFU/μg input DNA) on medium with ampicillin plus IPTG (Fig. 3a). Many more colonies (6.0 ± 5 × 10^4^ CFU/μg input DNA) grew when this transformation was plated on medium with ampicillin only (Fig. 3b). Re-ligated or undigested vector is expected to be most prevalent under these non-inducing conditions.

When assembling plasmids with longer inserts (>18 oligonucleotides), 5 ± 1 × 10^2^ to 4.6 ± 1 × 10^2^-CFU/ μg input DNA were obtained reproducibly on LB supplemented with ampicillin and IPTG. Screening by colony PCR (Fig. 2d–f and Table 1) and restriction profile analysis revealed that approximately half of the plasmids in this case were generated by vector re-circularization, 20–40% carried a randomly generated assembly, and 10–30% possessed an insert of the expected length. Thus, with more than 18 oligonucleotides simultaneously present in the mix, a drop in cloning efficiency was observed compared to when fewer oligonucleotides were used, with similar trends obtained for both the 24 and 28 oligonucleotide assemblies. Here, of the samples screened by PCR, 5 and 11% of the 24 and 28 oligonucleotide assemblies respectively, possessed a correctly sized insert (Fig. 2d, lanes 2 and 3, and Fig. 2e, lanes 3 and 8). Despite the reduced cloning efficiency with 24 and 28 oligonucleotide assemblies, more than half of these sequences were correct among samples selected by PCR (Table 1).

Overall the assembly of full-length and sub-fragments of the *aad1* gene demonstrated that *E. coli* can take up several annealed oligonucleotides and a linearized vector, as previously shown for the yeast *S. cerevisiae*^23^. In contrast to yeast, the successful cloning of gene-length fragments in *E. coli* required an *in vitro* pre-annealing step suggesting that the assembly of vector and insert in RapGene occurs mainly *in vitro*.

### RapGene assembly of the eukaryotic *gfp* gene

The *gfp* gene of *Aequorea victoria* encodes green fluorescent protein. The full-length *gfp* sequence (717 bp) was assembled in pRG1.0 by RapGene from 22 non-phosphorylated oligonucleotides (GFP-1 to GFP-22; Supplementary Table S1), and an additional 44 nt corresponding to the same ligation independent cohesive ends designed for cloning of *aadA1*. Thus, the complete *gfp* assembly spanned 761 bp. As for *aadA1*, no effort was made to optimize the oligonucleotides in order to avoid repeat sequences or to obtain comparable melting temperatures for the overlaps. The potential to improve the efficiency of RapGene by changing the assembly layout adopted for *aadA1* was examined (Table 1). Nineteen of the oligonucleotides used for *gfp* assembly were 66 nt and one each was 76, 80 and 68 nt (Supplementary Table S1) to accommodate the final construct in pRG1.0.

The average chemical transformation efficiency obtained from annealing of prepared pRG1.0 and *gfp* oligonucleotides was 4.8 ± 1 × 10^2^ CFU/μg input DNA, similar to that obtained for the full-length *aadA1* assembly. When 70 recombinant plasmids from different *gfp* cloning events were analysed by PCR, 20% possessed an insert of the expected size (Table 1). Sequencing of 14 of these latter plasmids carrying an insert of apparent correct length, showed that nine harboured the correct sequences corresponding to an accuracy of 64%. The frequency of correctly sized inserts for *gfp* (20%) was higher than for the 24–28 oligonucleotide assembly of *aadA1* (5–11%), but below that obtained for the 18 oligonucleotide construct for *aadA1* (73%). Overall these results suggest that several factors may affect RapGene assembly yields, and further work is needed to specifically investigate the effects of (i) variable oligonucleotide/overlap length, (ii) total number of oligonucleotides in the assembly and, as reported below, (iii) different oligonucleotide storage conditions. Formation of a full-length construct was also tested by assembling the *gfp* gene from smaller groups of consecutive oligonucleotides (GFP-1 to GFP-6, GFP-7 to GFP-13 and GFP-14 to GFP-22; Supplementary Tables S1 and S2) that were pre-annealed separately, and then pooled together with the prepared pRG1.0 before cloning. This approach did not lead to improvements over the standard protocol. *E. coli* NEB5α was the strain used throughout the study, but similar cloning efficiencies were obtained with the fast growing *E. coli* Turbo cells.

### Effect of freezing-thawing cycles on RapGene assembly

The effect of freezing-thawing cycles of oligonucleotide stocks on RapGene assembly was examined. Cloning efficiencies (as percentage of correct size inserts) with shorter *aadA1* subfragments (<18 oligonucleotides) were unaffected by two cycles of freezing at −20 °C followed by thawing to room temperature (Supplementary Table S2). In contrast, for longer assemblies the efficiency decreased sharply as oligonucleotides were subjected to increasing numbers of freezing-thawing cycles. For full length (28 oligonucleotides) and 24 oligonucleotides assembly of *aadA1*, these values changed from 20% after a single round of freezing-thawing to around 5% following four to six cycles (data not shown). Similarly, when pre-annealed assembly mixes containing 22 oligonucleotides for *gfp* assembly were subjected to a single cycle of freezing-thawing, the yields halved (Supplementary Table S2) but, as single use mixes were used, values stabilized at around 15–20%. Hence, degradation of single-stranded DNA during freezing-thawing cycles^26^,^27^ may be the cause for the lower efficiency observed with long assemblies here. At high concentration, degraded oligonucleotides with less than optimal overlaps may compete for and inhibit formation of the full length species.

### RapGene sequence accuracy and error rates

The most common types of sequence errors found in RapGene, among samples carrying an insert of apparent correct length, were deletions and insertions (Table 2) as reported for gene assembly in yeast^23^ and in a recent study^28^. It has previously been shown that *S. cerevisiae* can efficiently take up and assemble a mix of up to thirty-eight 60 nt oligonucleotides into gene sized fragments of 1.1–1.2 kb with a sequence accuracy up to 16.7%^23^. Although this value is lower than those observed here (64 and 57% for *gfp* and *aadA1*, respectively), the studies rely on different sample numbers and use genes of different lengths (up to 836 bp here vs up to 1170 bp with *S. cerevisiae*). Nevertheless, error rates in the two methods were compared by dividing the total number of errors observed, i.e., bases substituted, deleted or inserted, in all sequenced constructs by the total number of bases in these samples. Values were then multiplied by 100 as previously for gene assembly in yeast^23^. The error rate of 0.167 for RapGene (Table 2) is slightly lower than for an assembly using 38 oligonucleotides in *S. cerevisiae* (0.181)^23^. Although these values are quite similar, it cannot be excluded that the more stringent annealing conditions employed in RapGene may select against oligonucleotide mismatches and improve sequence accuracy as previously suggested for ligase chain reaction^6^.

## Discussion

Synthetic biology has provided powerful tools for the quick and extensive manipulation of DNA sequences of various length and complexity, thereby expanding the techniques made available by genetic engineering. Numerous tools are already available to the research community, and others are in development with the aim of tackling the limitations of current approaches and to further improve on the speed, accuracy and simplicity of DNA manipulation. Outsourcing options from gene synthesis companies are also becoming a more common practice in recent years, although it should be noted that the availability of such alternatives is a direct consequence of the continuous research efforts in the field.

In this paper a novel gene synthesis strategy was described in which an *in vitro/in vivo* approach was used to assemble selected genes from oligonucleotides and a linearized, positive selection vector into a recombinant plasmid in *E. coli*. A similar method was previously described for *S. cerevisiae*^23^ whose exceptional ability to recombine DNA fragments upon transformation was shown to be suitable not only for megabase assemblies, but also for gene synthesis applications. However, a drawback to that strategy was the intrinsic slow growth rate of yeast which produces visible colonies only after 3–4 days of incubation. The ability to conduct analogous assemblies in a fast growing species such as *E. coli* is clearly advantageous in terms of simplicity and speed. Although based on similar principles to the system described in yeast, RapGene employs *E. coli* as a cloning host with the entire process requiring no more than 24 hours using fast-growing strains. An additional benefit over the *S. cerevisiae* platform is that the recombinant plasmid obtained through RapGene does not need to be extracted from yeast and re-transformed in *E. coli* for sequencing or other purposes^23^. Instead colonies harbouring correct sequences can be used directly in downstream applications. As *E. coli* is a more tractable cloning host than yeast for molecular biology and synthetic biology applications, the technique described here provides a quick and easy alternative to existing protocols designed for gene assembly. Furthermore, once the vector has been prepared, RapGene, like the method of Gibson^23^, is an enzyme-free process, a feature which makes this approach simple, cheap and automation-friendly as compared with other protocols developed to date for gene assembly.

Optimization and improvements of existing DNA assembly methods were reported previously for techniques such as SLIC^29^, PCA^30^ and LCR^31^. SLIC was a development of LIC that allowed bypassing of strict DNA sequence requirements of the vector ends by omitting the addition of specific dNTPs to the exonuclease reaction step. Analogously, RapGene is an optimized assemblage of genetic parts and devices—some already described and some new—into an original composite system that comprises new properties and functions^32^, and which provides a biological platform for the rapid assembly and cloning of synthetic genes in *E. coli*. The method reported in this study has proven successful for synthesis and cloning of both prokaryotic and eukaryotic genes with an accuracy of about 1.7 errors per kilobase when employing unpurified (desalted) oligonucleotides. As indicated by Gibson’s study^23^ significantly improved accuracy is anticipated when using more highly purified oligonucleotides. It is also worth noting that the assembly of synthetic DNA with RapGene does not require electroporation but may be performed easily with chemically competent cells. The use of a positive selection vector further improves the efficiency and simplicity of the technique by avoiding the need for labour-intensive manipulations of the cloning plasmid. Nevertheless, use of a positive selection vector is not a strict requirement and RapGene potentially could be paired with alternative methods for the synthesis of recombinant DNA such as SliCE and SLIC. Coupled with SLIC, RapGene would already be an efficient strategy to re-design protein domains (between two SLIC-generated restrictions sites) up to approx. 600 bp in length with yield as high as 70%, as shown for the 18-oligonucleotide *aadA1* assembly. In this context, only selected oligonucleotides would be needed each time to generate mutants at specific positions, the others being re-usable in alternative assemblies. Vectors suitable for blue-white screening^33^ used in combination with RapGene are also convenient tools to quickly identify colonies carrying recombinant plasmids. However, as these vectors are not designed to reduce the number of background colonies, verification of complete plasmid digestion by gel electrophoresis remains a necessary step before ligation.

The RapGene and Gibson methods share similar features with SLIC as both are based on a ligation independent cloning approach. However, SLIC has been specifically described for assembly of multi-kilobase DNA fragments and not for gene synthesis applications. The starting material in SLIC consists of double-stranded DNA produced by PCR rather than single-stranded DNA, and each fragment of the final assembly needs to be gel extracted individually and purified before use. In contrast, oligonucleotides are employed directly in their unpurified form in RapGene to assemble the final construct. Finally, SLIC study does not address a particularly challenging aspect of gene synthesis which is the higher level of accuracy achievable when only chemically synthesized oligonucleotides are used. In fact, as the chemistry currently available for single-stranded DNA synthesis is not yet an error-free process, sequence accuracy is still a major issue in DNA fabrication technologies^22^,^28^.

Gene assembly here was performed using unpurified oligonucleotides. As yields for fully correct sequences were higher than those statistically predictable^34^, it is expected that RapGene can tolerate variable lengths of truncated single-stranded DNA species within the assembly mix (up to 40% for the ~60 nt oligonucleotides employed here). Whereas PCR-based gene synthesis methods should feature a similar behaviour, this may not be the case for techniques such as LCR in which the juxtaposition of consecutive oligonucleotides in the final construct is a fundamental requirement for ligation to occur^10^. Although oligonucleotide degradation due to repeated freeze-thawing cycles^26^,^27^ appears to be a limiting factor for RapGene with longer assemblies, improvements potentially can be achieved by use of alternative methods for DNA storage. In summary, RapGene provides a quick and accurate gene synthesis platform for the rapid design and assembly of synthetic genes or gene domains. The technique is suitable for diverse applications in synthetic biology at the bench level and above.

## Methods

### Enzymes, chemicals and media

Restriction enzymes, DNA polymerases, the Gibson Assembly Cloning Kit, dNTPs and SOC (Super Optimal broth with Catabolite repression) were purchased from New England Biolabs and were used following the manufacturer’s instructions. Luria-Bertani (LB) agar, ampicillin, spectinomycin and other chemicals were obtained from Sigma-Aldrich. Ampicillin and spectinomycin were used at 150 and 100 μg/ml, respectively. LB broth and agarose were purchased from Thermo Fisher Scientific and Melford, respectively. GelRed DNA dye was from Biotium Inc.

### Bacterial strains and plasmids

*E. coli* NEB5α and Turbo high efficiency, chemically competent cells were purchased from New England Biolabs. Plasmid pGATA^25^ positive selection vector was obtained from Purebiotech LLC and pRG1.0 was constructed as outlined below.

### Oligonucleotides, DNA ladders and DNA purification kit

Oligonucleotides (Supplementary Table S1) were purchased from Sigma-Aldrich at the lowest purification level (desalting), re-suspended in sterile 10 mM Tris-HCl pH 8.0, 0.1 mM EDTA, and stored at –80 °C. QIAprep Spin miniprep plasmid purification kit, QIAquick Gel Extraction Kit and QIAquick PCR Purification Kit were purchased from Qiagen, and the 100-bp and 1-kb DNA ladders were purchased from Promega.

### DNA quantification and sequencing

DNA concentrations were determined using the Qubit dsDNA BR Assay Kit (Life Technologies). Sequencing reactions were performed at GATC Biotech Ltd (London, UK).

### Colony PCR

The identification of plasmids carrying the correct inserts was done by PCR. Briefly, a single colony was picked from agar plates and re-suspended in 30 μl of dH_2_O in 0.2 ml tubes. The sample was heated at 96 °C for 5–10 minutes to lyse the cells, and 1 μl of this suspension was used as DNA template in 30 μl PCR reactions using Taq DNA polymerase.

### Construction of pRG1.0

Plasmid pGATA is a positive selection vector that includes a gene coding for the GATA-1 toxic peptide that inhibits bacterial growth when expression is induced from the upstream p*tac* promoter by IPTG. The peptide is produced as a glutathione-S-transferase (GST) fusion. Basal leaky expression of the toxic peptide is reduced to non-toxic levels by the simultaneous over-production of the LacI repressor whose gene (*lacI*^*q*^) is encoded on the same plasmid. Plasmid pRG1.0 (Supplementary Fig. S1) is a derivative of pGATA built from two PCR products amplified by Phusion DNA polymerase using primers PCR1f, PCR1r, PCR2f, and PCR2r (Supplementary Table S1). The plasmid was assembled from the PCR products (Supplementary Fig. S1) using the Gibson Assembly Cloning Kit. Primers were designed to introduce both restriction sites (NheI, AflII) and homology regions (26–30 bp end overlaps) required by the Gibson assembly method. Thus, pRG1.0 possesses NheI and AflII restriction sites flanking the toxic GST-*Gata1* (Supplementary Fig. S1). The sites were embedded into sequences suitable for ligation independent cloning to produce 21 nt (NheI side) and 23 nt (AflII side) 3’-overhangs upon treatment with T4 DNA polymerase and dTTPs (Supplementary Fig. S2). The spectinomycin promoter (p*Sm*) from plasmid pCDFDuet-1 (Merck-Novagen) was introduced upstream of the NheI site to drive expression of cloned synthetic genes. The region of pGATA downstream of *lacI* that comprised the first 147 codons of the *lacZ* gene was deleted to reduce the size of the plasmid. Colonies with candidate pRG1.0 plasmids obtained from this assembly technique were isolated on LB agar plates supplemented with ampicillin. Colonies subsequently were regrown overnight on duplicate LB agar plates supplemented with ampicillin with and without IPTG (1 mM). This step allowed identification of candidates with an IPTG-inducible *GST-Gata1* toxic element. Colonies growing on ampicillin plates, but not on ampicillin plates supplemented with IPTG, were cultured overnight and plasmids were extracted and digested with AclI. Plasmids with the expected restriction digestion profile were identified and the region between the *bla* N-terminus and *lacI*^*q*^ C-terminus that spans the cloning sites and the toxic element was sequence-verified. Due to the presence of the positive-selection marker derived from pGATA, pRG1.0 does not need gel extraction or other labour-intensive manipulations after restriction enzyme digestion, but instead can be purified by spin column procedures and used directly for gene assembly.

### RapGene procedure

#### Vector preparation

Plasmid pRG1.0 was prepared with long 3’-recessed cohesive ends to accommodate the annealed oligonucleotides. To this end, pRG1.0 was treated simultaneously with NheI, AflII and T4 DNA polymerase. Reactions were set up in buffer 2.1 (New England Biolabs) in the presence of 1 mM dTTPs and incubated at 37 °C for 1-2 hours in a heating block. A typical digestion was performed in 50–100 μl and contained 3–5 μg of plasmid DNA purified by standard miniprep procedures, 20 units of each restriction enzyme, and three units of T4 DNA polymerase. Reactions were cleaned up with a PCR purification kit, resuspended in 50 μl elution buffer, and stored at −80 °C or used directly for the next step of the assembly. DNA concentrations were in the range of 20–60 ng/ μl. Digestion of the vector was verified by agarose gel electrophoresis.

#### Insert preparation

The oligonucleotide assembly corresponding to the synthetic gene was prepared by slow annealing of six to 28 oligonucleotides (57–80 nt) covering the full-length of the sequence to be synthesized (Supplementary Table S1). Annealing reactions were performed in 1.5 ml tubes and consisted of 1 μl of each oligonucleotide (from 100 μM stocks prepared in sterile 10 mM Tris-HCl pH 8.0, 0.1 mM EDTA) and annealing buffer (10 mM Tris-HCl pH 8.0, 50 mM NaCl) in a final volume of 700 μl. For *gfp* only, single-use, pre-annealed oligonucleotide mixes were prepared and stored at −80 °C for later use, or employed directly for cloning without any freezing step. After vortexing, samples were incubated at 96 °C on a heating block which was then immediately turned off to allow the temperature to reach 30–35 °C (between 1.5 and 2 hours). The final DNA concentration of the full-length insert calculated as if all the oligonucleotides annealed into the correct assembly was approx. 140 nM (equivalent to that of each individual oligonucleotide in the mix).

### Annealing plasmid and insert

Prepared pRG1.0 vector and insert were assembled in a 0.2 ml tube. Typically the reaction consisted of approx. 0.015 pmol vector prepared as above, approx. 0.09 pmol pre-annealed oligonucleotide mix prepared as above, and annealing buffer (10 mM Tris-HCl, 50 mM NaCl, pH 8.0) in a final volume of 10 μl. This ratio of 1:6 vector:insert was optimal for number of transformants obtained when the full-length insert was assembled. The reaction was incubated at 45 °C on a heating block which was immediately turned off to reduce the temperature to 30 °C (45 minutes to 1 hour). 4-5 μl of the solution were mixed very gently with 50 μl chemically competent *E. coli* NEB5α or Turbo. Samples were incubated on ice for 30 minutes, heat shocked at 42 °C for 30 seconds, and then returned to ice immediately. After 2–5 minutes on ice 950 μl SOC were added, and this was followed by incubation at 37 °C for one hour. Transformations were plated on LB agar supplemented with ampicillin and IPTG. When assembling more than 18 oligonucleotides the whole transformation culture (1 ml) was plated, after pelleting and re-suspending it in 150 μl SOC.

### Designing the synthetic assembly

RapGene was tested successfully with the bacterial *aadA1* gene (792 bp; GenBank HQ880267.1) coding for aminoglycoside adenyltransferase that confers spectinomycin and streptomycin resistance, and the eukaryotic *gfp* gene (717 bp; GenBank L29345.1) coding for *Aequorea victoria* green fluorescent protein^35^. As the external oligonucleotides for both the synthetic assemblies contained single-stranded ligation independent cohesive ends (23 nt on the left and 21 nt on the right; Supplementary Fig. S2) designed for pRG1.0, the lengths of the synthetic elements spanned 836 bp (*aadA1*) and 761 bp (*gfp*). The *aadA1* gene was assembled and cloned in pRG1.0 from a total of 28 oligonucleotides (57–60 nt in length) (Supplementary Table S1), with 20 nt end-overlaps between consecutive oligonucleotides. 3’ truncations of *aadA1* were also assembled using RapGene to test the method with different length inserts. Five independent assemblies of increasing size were generated with this approach, the shortest consisting of the first six oligonucleotides from the 3’ end of *aadA1*, and the following increasing each by six additional consecutive oligonucleotides. The five different synthetic constructs made in this way were derived from 6, 12, 18, 24 and 28 oligonucleotides spanning 197, 371, 545, 719 and 836 bp, respectively. The synthetic *gfp* gene was assembled in pRG1.0 from 22 oligonucleotides each of which was 66 nt in length with 33 nt end-overlaps between consecutive oligonucleotides. In order to appropriately accommodate this insert into the vector used, the GFP2, GFP21 and GFP22 oligonucleotides (76, 80 and 68 nt, respectively) were designed to be longer than the other 19 oligonucleotides in the assembly. Gene design was planned manually for *aadA1*, or with the aid of Gene2Oligo software (http://berry.engin.umich.edu/gene2oligo)^36^ for *gfp*. No gaps were left between consecutive oligonucleotides in either the *aadA1* or *gfp* synthetic genes although, as reported for sequence and ligation independent cloning^20^ and from some preliminary results using RapGene (data not shown), any gaps between consecutive oligonucleotides are repaired efficiently *in vivo* by *E. coli*. Only two single nucleotide gaps were present on the vector during the assembly process in order to restore the restriction sites upon cloning. These two nucleotides corresponded to the first bases of NheI and AflII sites adjacent to the codons of the synthetic genes (Supplementary Fig. S2).

## Additional Information

**How to cite this article**: Zampini, M. *et al.* RapGene: a fast and accurate strategy for synthetic gene assembly in *Escherichia coli*. *Sci. Rep.* **5**, 11302; doi: 10.1038/srep11302 (2015).

## Supplementary Material

Supplementary Information

## Figures and Tables

**Table 1:** Cloning efficiency and sequence accuracy yields for the assembly of *gfp* (full length only) and *aadA1* (full length and subfragments) in pRG1.0.

***aadA1***												
Number of oligonucleotides in the assembly	Length of the assembly		Oligonucleotide lengths	Average overlaps within complementary pairs/ between consecutive pairs	CFU/μg LIC-treated pRG1.0 (mean ± s.d.)	Number of samples screened by colony PCR	Number of samples with correct-size insert	% of samples with correct-size insert	Number of samples sequenced	Number of samples with correct sequence	% of samples with correct sequence	Average CFU/plate normalized to 1 ml SOC overgrown culture (mean ± s.d.)
6	197 nt		57–60 nt	40/20 nt	>1.6 × 10^5^	30	29	97%	10	9	90%	>8000
12	371 nt		57–60 nt	40/20 nt	3.2 ± 6 × 10^3^	30	28	93%	10	7	70%	175 ± 15
18	545 nt		57–60 nt	40/20 nt	1.4 ± 2 × 10^3^	30	21	73%	10	3	30%	75 ± 5
24	719 nt		57–60 nt	40/20 nt	4.6 ± 1 × 10^2^	40	2	5%	2	2	100%	23 ± 5
28	836 nt		57–60 nt	40/20 nt	5 ± 1 × 10^2^	80	7	11%	7	4	57%	25 ± 5
***gfp***												
22	761 nt	33/33 nt	4.8 ± 1 × 10^2^	70	14	20%	14	9	64%	24 ± 5		

**Table 2:** Gene synthesis error rates and sources determined for RapGene. Data in the last four columns allow for a direct comparison with Gibson’s method^23^.

**Gene**	**Number of oligonucleotides**	**Bases sequenced**	**Number of bases deleted, inserted or substituted**	**Error rate**	**Number of bases deleted at each mutation event**	**Number of bases inserted at each mutation event**	**Number of bases substituted at each mutation event**	**Samples with 1 error**	**Samples with 2 errors**	**Samples with 3 errors**	**Samples with ≥4 errors**
*aadA1*	6	1970	1	0.050	0	0	1	1	0	0	0
*aadA1*	12	3719	3	0.080	1,1	1	0	3	0	0	0
*aadA1*	18	5446	20	0.367	2,4,5	1,1,2,3	1,1	2	1	0	7
*aadA1*	24	1438	0	0	0	0	0	0	0	0	0
*aadA1*	28	5016	2	0.039	1	1	0	0	0	1	1
*gfp*	22	9879	20	0.202	1,3,3,10	1,1,1	0	2	0	0	4
		Tot: **27468**	Tot:**46**		Tot:31	Tot:12	Tot:3				
		Final error rate:0.167 [(46/27468) x 100]			67.39% (31/46)	26.08% (12/46)	6.52% (3/46)				
