# Supplementary material for: RapGene: a fast and accurate strategy for synthetic gene assembly in Escherichia coli
Source: Sci Rep. 2015 Jun 11;5:11302. doi: 10.1038/srep11302 (PMC4462754; doi:10.1038/srep11302)
Supplement: Supplementary Information [file srep11302-s1.pdf]

Supplementary Information

RapGene: a fast and accurate strategy for synthetic gene assembly in *Escherichia coli*

Massimiliano Zampini, Pauline Rees Stevens, Justin A. Pachebat, Alison Kingston-Smith, Luis A. J. Mur & Finbarr Hayes

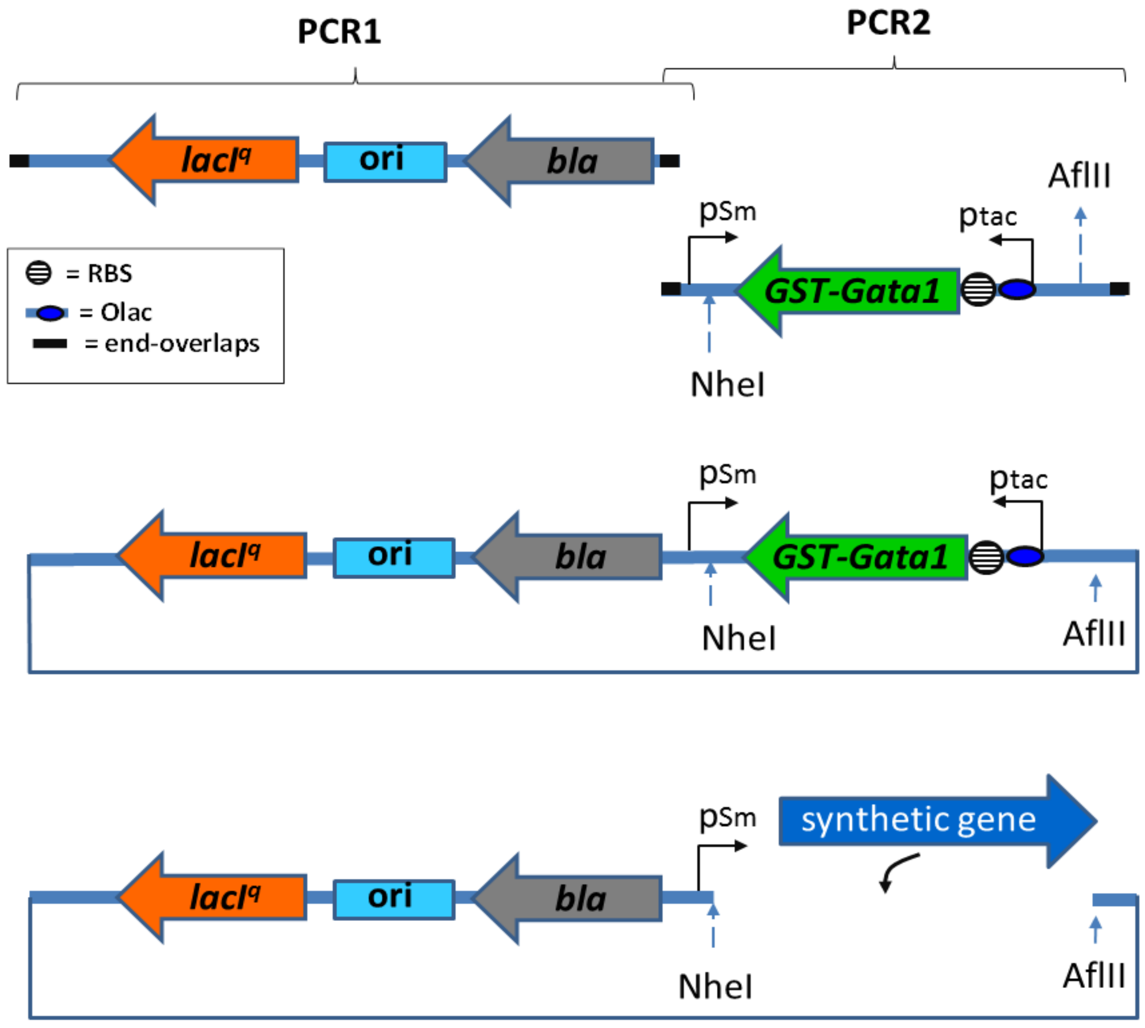

Supplementary Figure S1. pRG1.0 assembly and structure (not to scale). Plasmid pRG1.0 was assembled from two PCR products derived from pGATA using the Gibson Assembly Cloning Kit. pSm, streptomycin/spectinomycin promoter from pCDFDuet-1. ptac, tac promoter. RBS, ribosome binding site. Olac, lac operator. *bla*, beta-lactamase gene. ori, origin of replication. *lacI^q*, lac repressor gene with a modified promoter. NheI, restriction site for NheI. AflII, restriction site for AflII. End-overlaps, homology regions required by the Gibson assembly cloning kit.

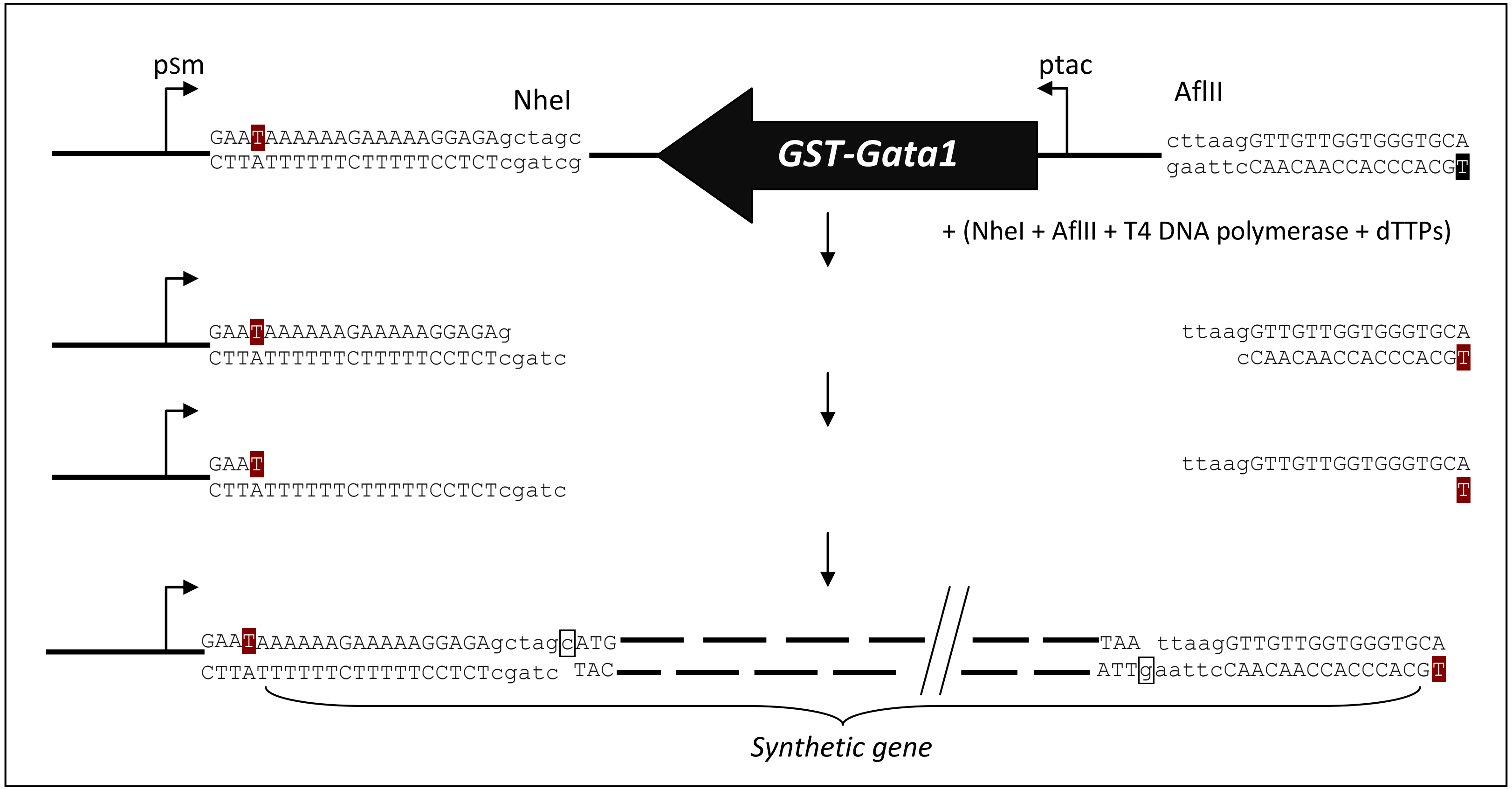

Supplementary Figure S2. pRG1.0 cloning region (not to scale). Plasmid pRG1.0 is prepared to generate long 3'-recessed cohesive ends by digesting with AflII and NheI in the presence of T4 DNA polymerase and 1 mM dTTPs. The reaction is stopped by spin-column purification. The single-nucleotide gap (boxed on the complementary strand) between the assembled synthetic insert and the vector, produced in all inserts after annealing, is filled *in vivo* in *E. coli*. Dashed lines represent the single oligonucleotides within the synthetic gene.

Name	Sequence (5’-3’)	Restriction sites	Signals	Notes
PCR1f	ACAGCTCActtaagGTTGTTGGTGGGTGCAGCCGGAAGCATAAAGTGT<br>AAAGCC	AflII		Template for PCR: pGATA
PCR1r	CCTAGGTTTATCAGGGTTATTGTCTCATGAGCGGATACATATTTGAAT<br>GTATTTAGAAAAATACCGGTACCTCTGACACATGCAGCTCCCGGAG		- promoter (-35 and -10 regions)	Template for PCR: pGATA
PCR2f	TCATGAGACAATAACCCTGATAAACCTAGGAATAAAAAAGAAAAAGG<br>AGAgctagcTTTCACCGTCATCACCGAAACGCGCGAGGCAGATCGTCA<br>G	NheI	- promoter (-10 region)	Template for PCR: pGATA
PCR2r	TGCACCCACCAACAACcttaagTGAGCTGTTGACAATTAATCATCGGCT<br>CG	AflII		Template for PCR: pGATA
Sm-1	AAAAAAGAAAAAGGAGAgctagcATGAGGGAAGCGGTGATCGCCGAAG<br>TATCGACTCAAC	NheI		top strand
Sm-2	GACGCCAACTACCTCTGATAGTTGAGTCGATACTTCGGCGATCACCG<br>CTTCCCTCAT			bottom strand
Sm-3	TATCAGAGGTAGTTGGCGTCATCGAGCGCCATCTCGAACCGACGTT<br>GCTGGCCGTACA			top strand
Sm-4	CCACTGCGGAGCCGTACAAATGTACGGCCAGCAACGTCGGTTCGAG<br>ATGGCGCTCGAT			bottom strand
Sm-5	TTTGTACGGCTCCGCAGTGGATGGCGGCCTGAAGCCACACAGTGAT<br>ATTGATTTGCTG			top strand
Sm-6	AGCCTTACGGTCACCGTAACCAGCAAATCAATATCACTGTGTGGCTT<br>CAGGCCGCCAT			bottom strand
Sm-7	GTTACGGTGACCGTAAGGCTTGATGAAACAACGCGGCGAGCTTTGAT<br>CAACGACCTTT			top strand
Sm-8	AGGGGAAGCCGAAGTTTCCAAAAGGTCGTTGATCAAAGCTCGCCGC<br>GTTGTTTCATCA			bottom strand
Sm-9	TGGAAACTTCGGCTTCCCCTGGAGAGAGCGAGATTCTCCGCGCTGT<br>AGAAGTCACCAT			top strand
Sm-10	TGATGTCGTCGTGCACAACAATGGTGACTTCTACAGCGCGGAGAATC<br>TCGCTCTCTCC			bottom strand
Sm-11	TGTTGTGCACGACGACATCATTCCGTGGCGTTATCCAGCTAAGCGCG<br>AACTGCAATTT			top strand

Sm-12	TCATTGCGCTGCCATTCTCCAAATTGCAGTTCGCGCTTAGCTGGATAACGCCACGGAA			bottom strand
Sm-13	GGAGAATGGCAGCGCAATGACATTCTTGCAGGTATCTTCGAGCCAGCCACGATCGACA			top strand
Sm-14	CAGCAAGATAGCCAGATCAATGTCGATCGTGGCTGGCTCGAAGATACCTGCAAGAATG			bottom strand
Sm-15	TTGATCTGGCTATCTTGCTGACAAAAGCAAGAGAACATAGCGTTGCCTTGGTAGGTCC			top strand
Sm-16	CAAAGAGTTCCTCCGCCGCTGGACCTACCAAGGCAACGCTATGTTCTCTTGCTTTTGT			bottom strand
Sm-17	AGCGGCGGAGGAACTCTTTGATCCGGTTCCTGAACAGGATCTATTTGAGGCGCTAAAT			top strand
Sm-18	TTCCATAGCGTTAAGGTTTCATTTAGCGCCTCAAATAGATCCTGTTCAGGAACCGGAT			bottom strand
Sm-19	GAAACCTTAACGCTATGGAACTCGCCGCCCGACTGGGCTGGCGATGAGCGAAATGTAG			top strand
Sm-20	AATGCGGGACAACGTAAGCACTACATTTCGCTCATCGCCAGCCCAGTCGGGCGGCGAG			bottom strand
Sm-21	TGCTTACGTTGTCCCGCATTTGGTACAGCGCAGTAACCGGCAAAATCGCGCCGAAGGA			top strand
Sm-22	TTGCCCAGTCGGCAGCGACATCCTTCGGCGCGATTTTGCCGGTTACTGCGCTGTACCA			bottom strand
Sm-23	TGTCGCTGCCGACTGGGCAATGGAGCGCCTGCCGGCCCAGTATCAGCCCGTCATACTT			top strand
Sm-24	AGATAAGCCTGTCTAGCTTCAAGTATGACGGGCTGATACTGGGCCGGCAGGCGCTCCA			bottom strand
Sm-25	GAAGCTAGACAGGCTTATCTTGGACAAGAAGAAGATCGCTTGGCCTCGCGCGCAGATC			top strand
Sm-26	GTGGACAAATTCTTCCAACTGATCTGCGCGCGAGGCCAAGCGATCTTCTTCTTGTCCA			bottom strand
Sm-27	AGTTGGAAGAATTTGTCCACTACGTGAAAGGCGAGATCACCAAGGTAGTCGGCAAATAA		- *aadA1* stop codon	top strand
Sm-28	GCACCCACCAACAACcttaagTTATTTGCCGACTACCTTGGTGATCTCGCCTTTCACGTA	AflII	- *aadA1* stop codon	bottom strand
Sm-A1	GCACCCACCAACAACcttaagCAGCAAATCAATATCACTGTGTGGCTTC	AflII		bottom strand

	AGGCCGCCAT			
Sm-A2	GCACCCACCAACAACcttaagAAATTGCAGTTCGCGCTTAGCTGGATAACGCCACGGAA	AflII		bottom strand
Sm-A3	GCACCCACCAACAACcttaagATTTAGCGCCTCAAATAGATCCTGTTCAGGAACCGGAT	AflII		bottom strand
Sm-A4	GCACCCACCAACAACcttaagAAGTATGACGGGCTGATACTGGGCCGGCAGGCGCTCCA	AflII		bottom strand
GFP-1	AAAAAAGAAAAAGGAGAgctagcATGAGTAAAGGAGAAGAACTTTTCACTGGAGTTGTCCCAATTC	NheI		top strand
GFP-2	TTTGCCCATTAACATCGCCATCTAATTCAACAAGAATTGGGACAACTCCAGTGAAAAGTTCTTCTCCTTTACTCAT			bottom strand
GFP-3	TTGTTGAATTAGATGGCGATGTTAATGGGCAAAAATTCTCTGTCAGTGGAGAGGGTGAAGGTGATG			top strand
GFP-4	AAATAAATTTAAGGGTAAGTTTTCCGTATGTTGCATCACCTTCACCCTCTCCACTGACAGAGAATT			bottom strand
GFP-5	CAACATACGGAAAACTTACCCTTAAATTTATTTGCACTACTGGGAAGCTACCTGTTCCATGGCCAA			top strand
GFP-6	ATTGAACACCATAAGAGAAAGTAGTGACAAGTGTTGGCCATGGAACAGGTAGCTTCCCAGTAGTGC			bottom strand
GFP-7	CACTTGTCACTACTTTCTCTTATGGTGTTCAATGCTTTTCAAGATACCCAGATCATATGAAACAGC			top strand
GFP-8	AACCTTCGGGCATGGCACTCTTGAAAAAGTCATGCTGTTTCATATGATCTGGGTATCTTGAAAAGC			bottom strand
GFP-9	ATGACTTTTTCAAGAGTGCCATGCCCGAAGGTTATGTACAGGAAAGAACTATATTTTACAAAGATG			top strand
GFP-10	ACTTGACTTCAGCACGTGTCTTGTAGTTCCCGTCATCTTTGTAAAATATAGTTCTTTCCTGTACAT			bottom strand
GFP-11	ACGGGAACTACAAGACACGTGCTGAAGTCAAGTTTGAAGGTGATACCCTTGTTAATAGAATCGAGT			top strand
GFP-12	TGTTTCCATCTTCTTTAAAATCAATACCTTTTAACTCGATTCTATTAACAAGGGTATCACCTTCAA			bottom strand
GFP-13	TAAAAGGTATTGATTTTAAAGAAGATGGAAACATTCTTGGACACAAAATGGAATACAACTATAACT			top strand
GFP-14	TTGGTTTGTCTGCCATGATGTATACATTATGTGAGTTATAGTTGTATTCCATTTTGTGTCCAAGAA			bottom strand

GFP-15	CACATAATGTATACATCATGGCAGACAAACCAAAGAATGGAATCAAA GTTAACTTCAAAATTAGAC			top strand
GFP-16	CTGCTAATTGAACGCTTCCATCTTTAATGTTGTGTCTAATTTTGAAGTT AACTTTGATTCCATTCT			bottom strand
GFP-17	ACAACATTAAAGATGGAAGCGTTCAATTAGCAGACCATTATCAACAAA ATACTCCAATTGGCGATG			top strand
GFP-18	ACAGGTAATGGTTGTCTGGTAAAAGGACAGGGCCATCGCCAATTGGA GTATTTTGTTGATAATGGT			bottom strand
GFP-19	GCCCTGTCCTTTTACCAGACAACCATTACCTGTCCACACAATCTGCC CTTTCCAAAGATCCCAACG			top strand
GFP-20	CAAACTCAAGAAGGATCATGTGATCTCTCTTTTCGTTGGGATCTTTGG AAAGGGCAGATTGTGTGG			bottom strand
GFP-21	AAAAGAGAGATCACATGATCCTTCTTGAGTTTGTAACAGCTGCTGGG ATTACACATGGCATGGATGAACTATACAAATAA		*gfp* stop codon	top strand
GFP-22	GCACCCACCAACAACcttaagTTATTTGTATAGTTCATCCATGCCATGTG TAATCCCAGCAGCTGTTA	AflII	*gfp* stop codon	bottom strand
RG1.0f	CTCCGGGAGCTGCATG			
RG1.0r	GGCACGACAGGTTTCCCG			

Supplementary Table S1. Oligonucleotides used in this study. Sequences designed to anneal to the DNA template in PCR are underlined. Boxed regions correspond to the homology sequences required by the Gibson Assembly Cloning Kit to produce pRG1.0. Sequences highlighted in black represents signals (i.e. promoters, stop codons). Restriction sites are indicated in lower case.

aadA1-assembly					
**Type of assembly**	***E. coli* strain**	**sample**	**Cycle 0**	**Cycle 1**	**Cycle 2**
Oligonucleotides annealed simultaneously	NEB5α	*aadA1* (6-oligonucleotides)	100%	100%	90%
Oligonucleotides annealed simultaneously	NEB5α	*aadA1* (12-oligonucleotides)	100%	100%	100%
Oligonucleotides annealed simultaneously	NEB5α	*aadA1* (18-oligonucleotides)	80%	70%	70%
Oligonucleotides annealed simultaneously	NEB5α	*aadA1* (24-oligonucleotides)	-	20%	-
Oligonucleotides annealed simultaneously	NEB5α	*aadA1* (28-oligonucleotides)	-	20%	10%
***gfp*-assembly**					
**Type of assembly**	***E. coli* strain**	**sample**	**Cycle 0**	**Cycle 1**	**Cycle 2**
Oligonucleotides annealed simultaneously	NEB5α	*gfp* (22-oligonucleotides)	44%	20%<br>15%<br>15%	-
Oligonucleotides pre-annealed separately in three groups	NEB5α	*gfp* (22-oligonucleotides)	-	20%<br>15%<br>10%	-

Supplementary Table S2. RapGene *aadA1* and *gfp* cloning efficiencies during the first two freezing-thawing cycles of the oligonucleotides used for the corresponding assemblies. Percentages of correct-size inserts as determined by colony PCR are reported. The table also reports yields for the assembly performed with three groups of consecutive oligonucleotides pre-annealed separately.
